# *MC1R* diversity in Northern Island Melanesia has not been constrained by strong purifying selection and cannot explain pigmentation phenotype variation in the region

**DOI:** 10.1186/s12863-015-0277-x

**Published:** 2015-10-19

**Authors:** Heather L. Norton, Elizabeth Werren, Jonathan Friedlaender

**Affiliations:** Department of Anthropology, University of Cincinnati, 481 Braunstein Hall, PO Box 210380, Cincinnati, OH 45221 USA; Department of Anthropology, 101 West Hall, University of Michigan, 1085 South University Ave, Ann Arbor, MI 48109 USA; Department of Anthropology, Temple University, Gladfelter Hall, 1115 West Berks Street, Philadelphia, PA 19122 USA

**Keywords:** Pigmentation phenotype, Natural selection, Island Melanesia

## Abstract

**Background:**

Variation in human skin pigmentation evolved in response to the selective pressure of ultra-violet radiation (UVR). Selection to maintain darker skin in high UVR environments is expected to constrain pigmentation phenotype and variation in pigmentation loci. Consistent with this hypothesis, the gene *MC1R* exhibits reduced diversity in African populations from high UVR regions compared to low-UVR non-African populations. However, *MC1R* diversity in non-African populations that have evolved under high-UVR conditions is not well characterized.

**Methods:**

In order to test the hypothesis that MC1R variation has been constrained in Melanesians the coding region of the MC1R gene was sequenced in 188 individuals from Northern Island Melanesia. The role of purifying selection was assessed using a modified McDonald Kreitman’s test. Pairwise F_ST_ was calculated between Melanesian populations and populations from the 1000 Genomes Project. The SNP rs2228479 was genotyped in a larger sample (n = 635) of Melanesians and tested for associations with skin and hair pigmentation.

**Results:**

We observe three nonsynonymous and two synonymous mutations. A modified McDonald Kreitman’s test failed to detect a significant signal of purifying selection. Pairwise F_ST_ values calculated between the four islands sampled here indicate little regional substructure in *MC1R*. When compared to African, European, East and South Asian populations, Melanesians do not exhibit reduced population divergence (measured as F_ST_) or a high proportion of haplotype sharing with Africans, as one might expect if ancestral haplotypes were conserved across high UVR populations in and out of Africa. The only common nonsynonymous polymorphism observed, rs2228479, is not significantly associated with skin or hair pigmentation in a larger sample of Melanesians.

**Conclusions:**

The pattern of sequence diversity here does not support a model of strong selective constraint on *MC1R* in Northern Island Melanesia This absence of strong constraint, as well as the recent population history of the region, may explain the observed frequencies of the derived rs2228479 allele. These results emphasize the complex genetic architecture of pigmentation phenotypes, which are controlled by multiple, possibly interacting loci. They also highlight the role that population history can play in influencing phenotypic diversity in the absence of strong natural selection.

**Electronic supplementary material:**

The online version of this article (doi:10.1186/s12863-015-0277-x) contains supplementary material, which is available to authorized users.

## Background

Human skin and hair pigmentation are highly variable traits that are controlled by multiple genetic loci [1–14]. Skin pigmentation in humans is tightly correlated with the intensity of ultra-violet radiation (UVR) [15]; darker pigmentation is commonly observed in populations originating from regions of higher UVR, while lighter skin color is common to populations in lower UVR regions. The geographic structure of human skin pigmentation variation strongly supports a model in which pigmentation phenotype has been influenced by natural selection [15–17]. It has been proposed that darker skin color in hominins evolved with the loss of body fur and hair, becoming established some time around 1.2 million years ago (mya) [18], presumably to protect against UV-induced damage to DNA and folic acid photolysis [19, 20]. Ultimately alleles causing lighter skin increased in frequency in populations expanding into lower UVR regions, possibly to increase the potential for vitamin D synthesis [15, 21].

Skin pigmentation is primarily determined by the amount, type, and distribution of melanin, one of the primary chromophores of the skin. Melanin (particularly the alkali-insoluble brown-black eumelanin) acts as both a barrier to and filter of UVR—it scatters UVR and limits its penetration of the epidermis [22, 23]. This photoprotective property takes on particular evolutionary significance in regions where UVR is high, as both the long (UVA) and short (UVB) wavelength radiation that reaches the earth’s surface have cytotoxic and mutagenic effects with potentially significant effects on fitness. UVB is absorbed by DNA, resulting in mutations such as cyclobutane pyrimidine dimers (CPDs) and pyrimidine (6–4) pyrimidine photoproducts [24, 25], both of which play a role in photocarcinogenesis [26]. The ability of melanin, particularly eumelanin, to minimize the potential cancer-causing properties of UVR has led some to speculate that darker skin pigmentation should be favored by natural selection in regions where UVR is high [20], although others argue that this is at best a weak selective force given the late age of onset of many fatal skin cancers [15, 16, 27, 28]. A perhaps more relevant evolutionary argument for the evolution and maintenance of a highly melanized skin in regions of high UVR is the need to minimize UVA mediated photolysis of the B-vitamin folate [19]. Folic acid is involved in DNA synthesis and repair as well as spermatogenesis [29, 30], and folic acid deficiencies in reproductive-age females have been linked to an increased risk of neural tube birth defects [31, 32]. As with the photoprotection hypothesis, the folic acid hypothesis predicts that darker skin color should be maintained by purifying selection in high UVR regions [15, 16, 33].

A genetic locus known to influence human skin and hair pigmentation is the *MC1R* gene, which encodes the melanocortin-1 receptor, a 7-pass transmembrane G-protein coupled receptor found on the surface of melanocyte cells. When the hormone α-MSH binds to the MC1R, activation of adenyl cyclase results in increased levels of cAMP. This leads to an increase in the activity of tyrosinase, the rate-limiting enzyme of melanogenesis, as well as increased levels of tyrosinase-related proteins (TRP)-1 and −2 [34, 35], ultimately resulting in eumelanin synthesis. However, if instead the antagonist agouti-signaling protein binds to the MC1R, pheomelanin production results. Despite its small size (951 bp), the *MC1R* gene harbors a high number of polymorphisms, including several loss-of-function mutations that are associated with reduced skin color, melanoma, freckling, and red or blond hair [7, 36–39]. *MC1R* mutations are also associated with a reduced DNA-repair capacity, possibly explaining the link between *MC1R* and melanoma risk [40, 41].

*MC1R* has received particular attention in studies of human evolution because of its unusual levels and patterns of sequence diversity. Unlike many loci, which exhibit higher levels of diversity in African populations, *MC1R* diversity is highest in Eurasian populations [42, 43]. The lower sequence diversity observed in Africans is commonly attributed to purifying selection, while the higher diversity in Eurasian populations has been alternatively interpreted as being due to either relaxed functional constraint or positive selection for lighter skin color [42, 43]. It has been suggested that the higher diversity observed at *MC1R* can be attributed in part to a high mutation rate related to the elevated CpG content of the region [37]. This makes the reduced diversity in high-UVR African populations all the more notable.

Evidence for purifying selection on *MC1R* in African and other high-UVR populations rests primarily on low levels of nucleotide diversity and the ratios of nonsynonymous to synonymous polymorphisms and divergent sites (assessed using McDonald-Kreitman and HKA tests) [42–44]. The higher sequence diversity observed at *MC1R* in non-African populations has been argued to be a signal of diversifying selection [43], although this is not supported by McDonald Kreitman and HKA tests [42]. Tests of natural selection that rely on the site frequency spectrum, inter-population divergence, and extended haplotype homozygosity are also in conflict as to the nature of selection acting on *MC1R* in non-African populations [10, 37, 42, 45, 46].

Because of its association with several phenotypic traits in European (and to a lesser extent East Asian) populations, *MC1R* has been extensively sequenced in populations living in low UVR regions. However, far less is known of *MC1R* sequence diversity in high UVR populations outside of Africa, including whether or not polymorphisms segregating in such populations may be responsible for variation in skin or hair pigmentation phenotype in these regions. Because *MC1R* is commonly associated with mutations that lead to a *decrease* in skin pigmentation, it is generally assumed that *MC1R* variation is tightly constrained by purifying selection in high UVR regions. However, it is possible that mutations leading to an *increase* in the synthesis of eumelanin may have been favored by positive selection. While such mutations in *MC1R* are believe to have played an important role in adaptation following the loss of body hair in hominins 1.2 mya [18], there is little evidence to date for more recent mutations with this effect occurring in humans. As such, most investigations of selection acting on *MC1R* in high-UVR populations focus on the role of purifying, rather than positive, selection.

Early reports of sequence variation in *MC1R* indicated that the gene was under strong functional constraint in populations from Papua New Guinea as well as Africa, as one might expect if purifying selection has acted to remove nonsynonymous variants resulting in lighter skin color [42, 43] across high UVR regions. However, those studies sampled only a small number of Melanesians (32 chromosomes) [42], resulting in a limited picture of *MC1R* diversity in high-UVR non-African populations. In order to accurately assess the extent of variation at *MC1R* in Melanesia, a broader sample is critical, due to the complex population history of the region [47–51]. Archaeological evidence indicates that modern humans reached Near Oceania (Sahul, the New Guinea and Australia landmasses, the Bismarck Archipelago, and much of the Solomon islands) by 49,000 YBP [52, 53], spreading as far east as the island of Buka in the Solomons by 29,000 YBP [54]. The region later saw a major influx of migrants speaking languages belonging to the Proto-Oceanic Austronesian language family around 4 KYA [55, 56] originating from a homeland in Taiwan. Thus, while *MC1R* variation is expected to have been tightly constrained during much of the first ~30,000–40,000 years of human habitation in Melanesia, the migration of Austronesian speakers into the region and their subsequent admixture with resident populations may have introduced nonsynonymous mutations more commonly found in low-UVR regions. If not quickly removed by strong purifying selection such variants may contribute to observed variation in pigmentation phenotype.

Populations from Island Melanesia are darkly pigmented compared to populations of northern Europe and Asia [57], as expected if pigmentation phenotype reflects adaptation to UVR intensity [15]. However, despite experiencing high levels of UVR, Northern Island Melanesian populations exhibit a striking amount of variation in skin pigmentation [58]. Perhaps even more unusually, some Melanesians also exhibit a characteristic “blond hair” phenotype that is observed from Northern Island Melanesia throughout the Solomon Islands, which can be partially explained by a nonsynonymous variant in the *TYRP1* gene [58, 59]. Variation in skin and hair pigmentation is particularly pronounced between different islands of Northern Island Melanesia, including the island of Bougainville (located at the northwest tip of the Solomon Islands chain) and islands in the Bismarck Archipelago. It is highly unlikely that this variation is due to very fine scale adaptation to UVR differences in the region [58]. Instead, this variation suggests that even in a high-UVR environment pigmentation phenotype may vary so long as it is maintained above a protective melanin “threshold” [58, 60]. *MC1R*, known to influence skin and hair color in European and East Asian populations [1, 36, 38, 39, 61, 62] is a possible candidate to explain a portion of this observed variation.

Here we survey sequence variation in the coding region of the *MC1R* gene in 188 Island Melanesians from four different islands: New Hanover, New Britain, New Ireland, and Bougainville. To our knowledge this is the most extensive survey of *MC1R* sequence variation in the region to date. We use these data to address three questions pertaining to the evolution of *MC1R* sequence variation in Northern Island Melanesian populations and the role of *MC1R* in shaping phenotypic diversity in the region. Specifically we set out to test the hypothesis that variation in *MC1R* has been constrained in these populations by *purifying* selection. We also characterized regional (inter-island) and global levels of variation at *MC1R* in order to assess the roles of selection and population history in shaping variation at *MC1R* in Northern Island Melanesia. Finally, we tested for associations between *MC1R* polymorphisms and quantitatively measured skin and hair pigmentation phenotype to evaluate the role of *MC1R* in shaping local pigmentation phenotype.

## Methods

### Sample collection

**Fig. 1:**
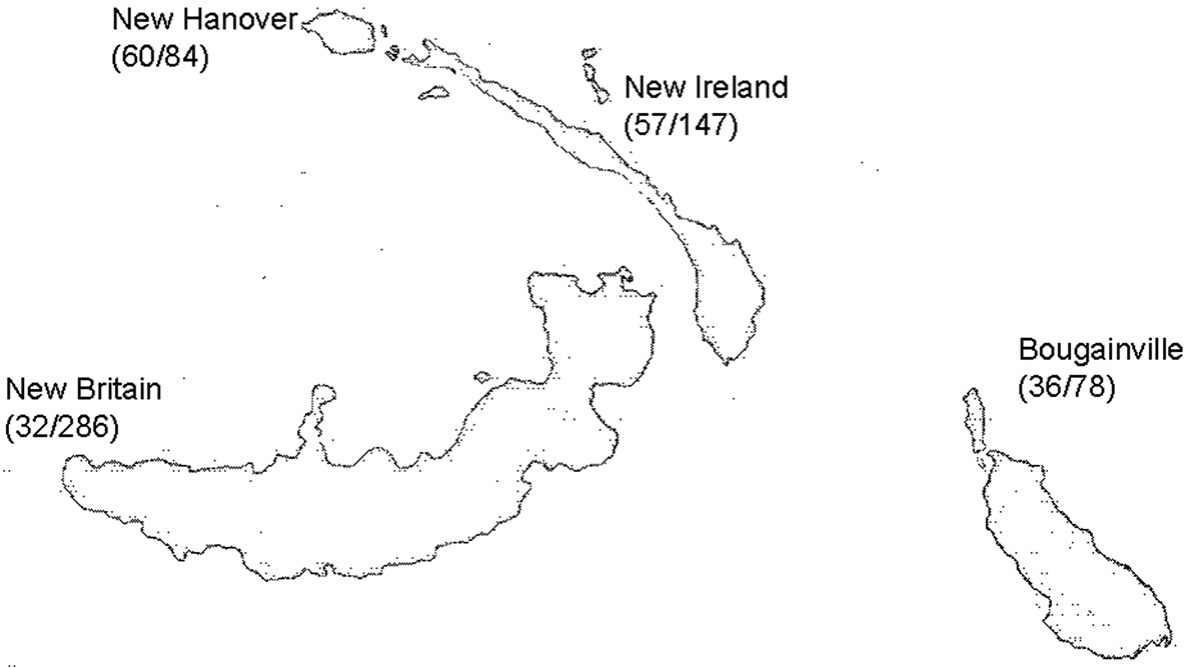
Map of study region, highlighting the four main islands sampled (New Hanover, New Britain, New Ireland, and Bougainville). Numbers next to each island represent the total number of individuals sequenced and genotyped from each island

The pigmentation measurements reported here were originally collected as part of a larger study examining phenotypic variation and population history in Island Melanesia by H.L.N. and J.S.F conducted in 2000 and 2003. Individuals were sampled from islands throughout Northern Island Melanesia, with an emphasis on the islands of New Britain, New Hanover, New Ireland, and Bougainville (Fig. 1). Individuals speaking languages belonging to the Oceanic division of the Austronesian (AN) phylum as well as speakers of unrelated non-Austronesian (Papuan) languages were recruited [50]. In total 1135 adults were sampled, of which 188 were used here in DNA sequencing and an additional 444 in the genotyping of the rs2228479 SNP. DNA samples were chosen for sequencing and genotyping based on the following factors: the quantity and quality of available DNA, representation of all four major islands and two linguistic phyla in the region, and, where possible, an attempt to focus heavily on 1–3 neighborhoods or subpopulations within an island (in an effort to minimize intra-island substructure).

Individuals were assigned to categories according to sex, island, neighborhood, and linguistic phylum. In order to be assigned to a particular island, neighborhood, or linguistic phylum an individual and both of his or her parents needed to also be from that island/neighborhood or speak a language belonging to the same phylum. All individuals gave their informed consent to participate in the study, including the measurement of pigmentation data and the examination and publication of genetic variation. Institutional Review Board (IRB) approval for data collection and analyses were obtained from Temple University (IRB 99–226), The Pennsylvania State University (IRB 00 M558-2), and the Papua New Guinea Medical Research Advisory Committee. Approval was granted for all data collection sites. This was a collaborative project with the PNG Institute of Medical Research.

### Pigmentation measurement

Quantitative measurements of skin and hair pigmentation were taken using the DermaSpectrometer (Cortex Technology, Hadsund, Denmark) following the practices described in Norton et al. [58]. The DermaSpectrometer estimates the concentrations of the two primary chromophores of the skin, hemoglobin and melanin, and reports this as the melanin (*M*) index. The *M* index provides a quantitative measure of skin color that is due primarily to the effects of melanin alone [63].

### *MC1R* sequencing and genotyping

The coding region of the *MC1R* gene (951 bp) was amplified in the 191 Melanesian samples from the islands of Bougainville (*N* = 36), New Hanover (*n* = 60), New Britain (*n* = 32), and New Ireland (*n* = 57). Sequence was also obtained from three individuals who could not be clearly assigned to any one of these four islands (see *Sample Collection*). Product amplification was verified using gel electrophoresis, and amplified products were purified for sequencing using the GeneJet Purification kit (Life Technologies). Sequence data were aligned to the human reference sequence using the Geneious 6.0 software package [64]. Variants were identified using the “Find Heterozygotes” feature in Geneious and confirmed by visual inspection. Haplotypes were computationally phased using the program Phase 2.1.1 [65], and all phases were correctly inferred (*p* > 0.85) Melanesian sequences were later aligned with the chimpanzee (*Pan troglodytes*) *MC1R* sequence and African (LWK, YRI), East Asian (CHB, CHS, JPT), European (CEU, FIN, GBR, IBS, and TSI), and South Asian (BEB, GIH, ITU, PJL, STU) samples sequenced as part of the 1000 Genomes Project (http://www.1000Genomes.org, accessed on August 28, 2014).

In order to test for the effect of the derived allele at rs2228479 on skin and hair pigmentation in a larger sample of Melanesians we utilized published genotype and phenotype data from these individuals [66]. To obtain genotype data on additional individuals we designed separate amplification primers (available from the authors on request) to amplify a 695 bp region around rs2228479. The restriction enzyme NspI, which recognizes the derived allele, was used to digest these amplified fragments under standard conditions. Digested products were visualized using gel electrophoresis.

### Statistical analyses

Summary statistics for *MC1R* sequence diversity and polymorphism and divergence were calculated in DNASp v5.10.1 [67, 68]. Two measures of nucleotide diversity were calculated: π [69], which is based on the average number of nucleotide differences between two sequences randomly drawn from a sample; and θ, which is based on the proportion of segregating sites in the sample [70]. Tajima’s D, a summary of the allele frequency spectrum that tests the null hypothesis of mutation-drift equilibrium and constant population size (under which π and θ are approximately equal) was also calculated [71]. A negative value of Tajima’s D indicates an excess of rare alleles, which may occur when a population is growing or when a gene is targeted by purifying selection. Positive values may be indicative of a population bottleneck or balancing selection. We assessed statistical significance of observed Tajima’s D values by comparing them to values obtained from 10,000 simulations under a standard neutral model. Because demographic history (e.g. a population bottleneck) can mimic the effects of selection, we also compared observed values in the Melanesian sample those obtained under 10,000 simulations of a simple bottleneck model marking the divergence of Melanesian populations from an ancestral Eurasian population. As no well-defined demographic model has yet been developed to characterize the population history of the five populations used here (the four 1000 Genome populations and the Melanesians), we use bottleneck and divergence parameters estimates for a New Guinea Highlands population estimated by Wollstein et al. [72]. This model is designed primarily to capture the reduction in diversity associated with the initial colonization of Melanesia.

In order to test the hypothesis that *MC1R* has been shaped by purifying selection in Island Melanesian populations we use a modification of the McDonald-Kreitman test, which compares the ratio of nonsynonymous to synonymous polymorphisms to the ratio of nonsynonymous to synonymous fixed differences [73]. An advantage of using the McDonald-Kreitman test over other site-frequency spectrum based tests (such as Tajima’s D and Fay and Wu’s H) is that it is relatively insensitive to variation in demographic history [74, 75]. Due to the small number of segregating sites that we observe in the Melanesian sample (five), we follow Harding et al. [42] and use an exact test described by Sokal and Rohlf (1969). In this test, we use the number of fixed nonsynonymous (ten) to synonymous (six) changes between humans and chimpanzee, and assume that new nonsynonymous and synonymous mutations will occur with a binomial probability of 0.625 and 0.375, respectively [42]. The sum of probabilities for the observed ratio of nonsynonymous (*n* = 3) to synonymous (*n* = 2) polymorphisms in Melanesians as well as for all other equally or less likely probabilities provides a test of this null hypothesis.

Allele frequency distinctions between islands and between linguistic phyla were tested for using a *χ*^2^ test implemented in DNASp [67]. Tests of Hardy Weinberg Equilibrium for each Melanesian island were performed in PLINK [76]. Pairwise F_ST_ values for the coding region of *MC1R* were calculated using DNASp between each of the four Melanesian islands, between each island and each population from the 1000 Genomes Project, and between the Melanesian region as a whole 1000 Genomes African, East Asian, European, and South Asian regional groupings. Median-joining haplotype networks showing the relationship of *MC1R* haplotypes among Melanesian islands and between Melanesians and the 1000 Genomes samples were constructed using Network 4.613 [77].

Associations between the only common (frequency > 0.05) *MC1R* nonsynonymous polymorphism, rs2228479, and skin and hair pigmentation in the Melanesian population were tested for in PLINK [76] using linear regression with an additive model. We tested for associations in the full Melanesian sample using island as a covariate in an effort to control for inter-island substructure in the region [48]. We also tested for associations on each island separately. Due to the skewed distribution of the pigmentation measurements, skin and hair M index values were first transformed using a Box-Cox procedure (skin *M* λ = −0.788, hair *M* λ = 2.93). The distribution of untransformed and transformed skin and hair M index values are depicted in Additional files 1 and 2.

## Results

### MC1R sequence variation

In our sample of 188 sequenced Melanesians, we observed a total of five segregating sites: three of these were synonymous mutations, two were nonsynonymous. Of these five mutations, one (a nonsynonymous Ile → Leu mutation at position 89986456) is novel and has not been previously reported. However, this mutation is rare in the sample, occurring in three individuals (all from the island of New Hanover). The most common *MC1R* mutations observed here are rs2228479 (V92M), rs885479 (R163G), and rs2228478 (T314T), which occur at frequencies of 15.4, 4.5, and 22.3 % in the resequenced sample, respectively. *MC1R* haplotypes and their frequencies in the Melanesian sample are reported in Table 1.

**Table 1:** Melanesian *MC1R* haplotypes and total number of observed haplotypes for each island and linguistic phylum. Note—because some individuals could not be assigned to an island and/or phylum, the sum of the haplotypes for a given island/phylum may not equal the total number of observed haplotypes in the total Melanesian sample

	rs22228479	Rs372929572	rs885479	Ile → Leu	rs2228478	Island				Languages		Total Melanesian
HAPLOTYPE #	89985940	89986083	89986154	89986456	89986608	Bougainville	New Hanover	New Britain	New Ireland	Austronesian	Papuan	
1	G	C	A	A	A	0	5	6	6	16	1	17
2 (human consensus)	G	C	G	A	A	57	69	45	82	155	99	258
3	A	C	G	A	A	0	9	3	2	12	2	14
4	A	C	G	A	G	8	22	4	9	38	5	43
5	G	C	G	A	G	7	13	6	12	31	8	39
6	G	T	G	A	A	0	0	0	0	0	0	2
7	G	C	G	C	G	0	1	0	1	2	0	2
8	G	C	G	C	A	0	1	0	0	1	0	1

We calculated average nucleotide diversity (measured as π and θ) in the total Melanesian sample, as well as for each island and linguistic phylum separately. These are displayed in Table 2, with values for Africans, East Asians, Europeans, and South Asians sequenced by the 1000 Genomes Project included for comparison. π in the full Melanesian sample (0.00075) was lower than that observed for the AFR, EAS, and EUR regional samples. However, it was higher than the observed value for the SAS samples (0.00061). The Melanesian θ value (0.00081) was less than the reported value for all four 1000 Genomes population samples. Thinking that the lower diversity levels observed in the Melanesian sample might reflect the smaller sample size of the Melanesians (2 *N* = 376) compared to the larger regional 1000 Genome populations (2 N range: 370–758), we randomly subsampled all regional populations (MEL, AFR, EAS, EUR, and SAS) to obtain 370 haplotypes (the total number in the smallest, AFR, sample) from each and recalculated summary statistics. This process was repeated 100 times to obtain a range of π and θ values for each subsampled population (see Additional file 3: Table S1). As expected, subsampling leads to a reduction in diversity for for both π and θ in all five populations, with Melanesian diversity at both π and θ remaining low. Mean π is lower in the subsampled Melanesian sample than in the subsampled South Asian sample, and mean θ in the subsampled Melanesians is the lowest of all the subsampled population values.

**Table 2:** Summary statistics for *MC1R* in Melanesian sample and for 1000 Genomes populations (AFR = LWK, YRI; EUR = CEU, FIN, GBR, IBS, TSI; EAS = CHB, CHD, JPT; SAS = BEB, GIH, ITU, PJL, STU)

Population	2 N	bp	S	# of haplotypes	π	θ	Tajima’s D	FWH
Melanesia	376	954	5	8	0.00075	0.00081	−0.117	−0.543
Bougainville	72	954	2	3	0.00056	0.00043	0.486	−0.761
New Hanover	122	954	5	7	0.00099	0.00097	0.036	−0.191
New Britain	64	954	3	5	0.00067	0.00067	0.012	−0.850
New Ireland	112	954	4	6	0.00065	0.00079	−0.361	−0.715
Austronesian speaking	246	954	4	7	0.00087	0.00069	0.445	−0.362
Non-Austronesian speaking	124	954	3	5	0.00050	0.00058	−0.249	−0.934
AFR	370	954	10	13	0.00086	0.00162	−1.019	0.160
LWK	194	954	9	10	0.00082	0.00161	−1.12	0.123
YRI	176	954	7	9	0.00090	0.00128	−0.631	0.193
EUR	758	954	17	18	0.00104	0.00247	−1.338	−2.749
CEU	170	954	9	10	0.00101	0.00165	−0.902	−2.866
FIN	186	954	11	11	0.00107	0.00199	−1.109	−0.863
GBR	178	954	10	11	0.00122	0.00182	−0.778	−0.437
IBS	28	954	4	5	0.00054	0.00108	−1.30	−1.481
TSI	196	954	14	15	0.00087	0.00251	−1.65	−0.898
EAS	572	954	8	10	0.00138	0.00121	0.269	−0.802
CHB	194	954	6	6	0.00142	0.00108	0.657	−0.787
CHD	200	954	5	5	0.00157	0.00089	1.451	−0.285
JPT	178	954	6	8	0.00098	0.00109	−0.220	−1.697
SAS	978	954	23	24	0.00061	0.00323	−1.939	−2.662
BEB	172	954	7	8	0.00070	0.00128	−0.989	−0.611
GIH	206	954	13	14	0.00059	0.00231	−1.833	−0.635
ITU	204	954	12	13	0.00064	0.00213	−1.700	−2.672
PJL	192	954	10	11	0.00060	0.00180	−1.562	−0.677
STU	204	954	9	10	0.00056	0.00160	−1.479	−0.741

Mean levels of diversity and haplotype distribution across the four Melanesian islands sampled here can be found in Tables 1 and 2. Of the four islands, diversity is lowest on Bougainville (π = 0.00059, θ = 0.00043), where only two segregating sites and three haplotypes are present. Diversity levels are highest on New Hanover (π = 0.00099, θ = 0.00097), where the highest number segregating sites (five) and haplotypes (seven) are observed. Diversity values for each of the four Melanesian islands also indicate relatively low levels of variation compared to the individual 1000 Genomes populations. Notably π values in non-Austronesian speakers (π = 0.00050) are roughly one-half of those reported for Austronesian speakers (π = 0.00087), indicating a sharp reduction in diversity among non-Austronesians.

### Evidence for purifying selection

Given the ten fixed nonsynonymous and six fixed synonymous changes between humans and chimpanzees, we estimate the likelihood of observing three nonsynonymous polymorphisms out of five total segregating sites to be 0.275. These data do not support a model in which variation in *MC1R* has been tightly constrained by strong purifying selection in Melanesian populations. Although site-frequency-spectrum based statistics such as Tajima’s D and Fay and Wu’s H are sensitive to demographic history, we also calculated values for these statistics in the full Melanesian sample, as well as for each individual Melanesian population (Table 2). Tajima’s D is slightly negative in the population samples from New Ireland and in non-Austronesian speakers, while it is slightly positive on the remaining islands and in Austronesian speakers Tajima’s D in the full Melanesian sample was −0.117. None of these values fall outside the 95 % confidence intervals of Tajima’s D values obtained from simulations under either the standard neutral (−1.412 – 1.696) or bottleneck model (−0.911 – 2.932). Fay and Wu’s H is slightly negative in all Melanesian populations sampled (−0.850 – -0.191). As with Tajima’s D, none of these values are outside the 95 % confidence limits estimated for either the standard neutral (−2.857 – 1.349) or bottleneck models (−2.986 – 1.014).

### Inter-population variation

**Fig. 2:**
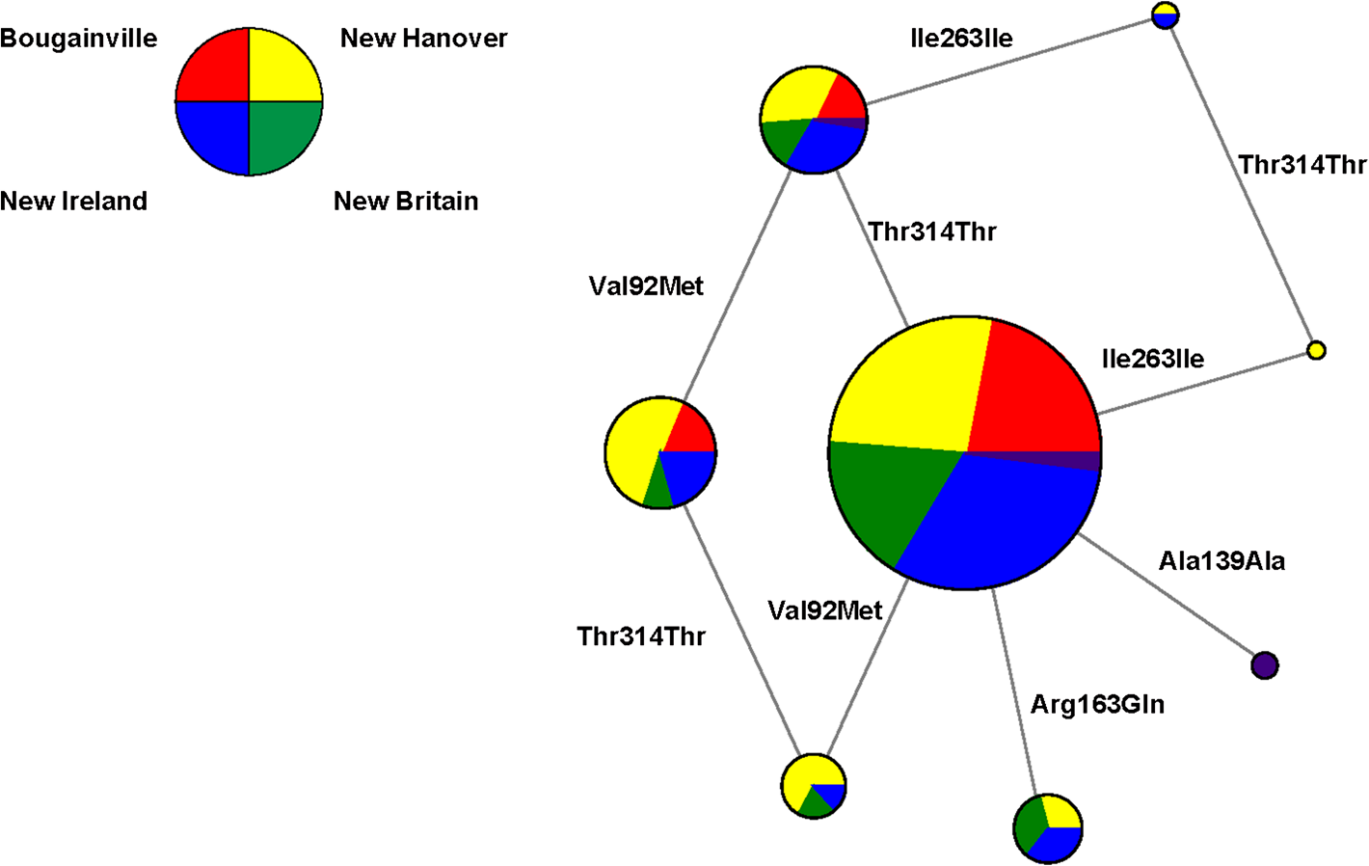
*MC1R* haplotype network of Melanesian samples, colored according to island (Bougainville: red; New Hanover: yellow; New Britain: green; New Ireland: blue; unaffiliated island: gray). Size of the each circle is proportional to the frequency of that haplotype. HCS: Human Consensus Sequence

Among the four Melanesian islands sampled here, pairwise F_ST_ values at *MC1R* are generally low (0.000–0.042), with the highest values reported for comparisons between New Hanover and New Britain (0.042), and the lowest between New Ireland and Bougainville (0.000) and between New Ireland and New Britain (0.000). By comparison, pairwise F_ST_ values at *MC1R* among the five European, two African, three East Asian, and five South Asian populations in the 1000 Genomes Project ranged from 0.001–0.065 (European) 0.000 (African), 0.022–0.089 (East Asian), and 0.000–0.004 (South Asian) (see Additional file 3: Table S2). Among the eight *MC1R* haplotypes observed in the Melanesian sample, three of the five common haplotypes (haplotype frequency > 1 %) can be found on each of the 4 islands (Table 1, Fig. 2), also indicating relatively little regional substructure at *MC1R*.

**Fig. 3:**
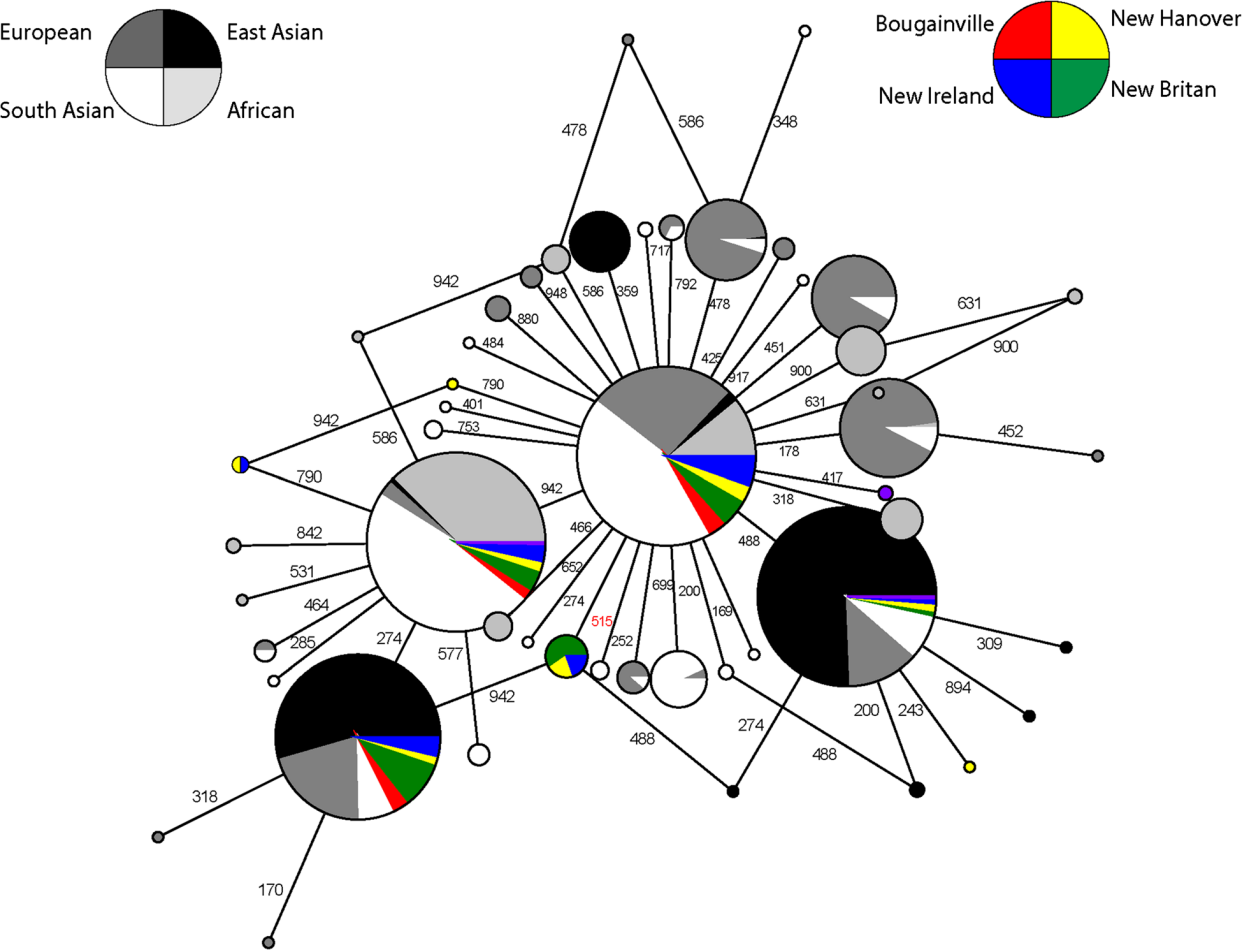
*MC1R* haplotype network of Melanesian and 1000 Genomes samples. Melanesian haplotypes are colored according to island (Bougainville: red; New Hanover: yellow; New Britain: green; New Ireland: blue; unaffiliated island: purple) and 1000 Genomes haplotypes are shown in grayscale (African: light gray; East Asian: black; European: dark gray; South Asian: white). The size of the each circle is roughly proportional to the frequency of that haplotype. Numbers next to mutational branches refer to the base position in the *MC1R* coding sequence where the mutation occurs. Base position 515, the location of rs2228479 is highlighted in red

Pairwise F_ST_ values between the full Melanesian sample and the 1000 Genomes AFR, EUR, EAS, and SAS regional population samples are found in Table 3. Surprisingly, Melanesians exhibit the lowest pairwise F_ST_ values with South Asians (F_ST(MEL-SAS)_ = 0.027) and the highest with East Asians (F_ST(MEL-EAS)_ = 0.270). However, this high divergence between Melanesians and East Asians is characteristic of generally high levels of divergence between East Asians and the other 3 populations at *MC1R* (F_ST_ values range between 0.255 and 0.326 for all East Asian-specific F_ST_ comparisons). There is no evidence of a particularly strong affinity between Melanesians and the high-UVR African populations of the 1000 Genomes Project (Table 3, Fig. 3). The majority of common (>1 %) Melanesian *MC1R* haplotypes are found in Melanesians as well as Africans, East Asians, Europeans, and South Asians Of the eight haplotypes observed in the Melanesian sample, none are shared exclusively with any of these groups (Fig. 3). When pairwise F_ST_ is calculated between each Melanesian island and the individual 1000 Genome populations there is little evidence to suggest particularly low levels of divergence between a specific island and any individual 1000 Genomes population (see Additional file 3: Table S2).

**Table 3:** Pairwse F_ST_ of *MC1R* between Melanesian and 1000 genomes regional populations

AFR	0.088			
EUR	0.054	0.148		
EAS	0.270	0.326	0.255	
SAS	0.027	0.075	0.048	0.306
	MEL	AFR	EUR	EAS

### rs228479 frequency and associations with phenotype across Melanesia

The only common nonsynonymous SNP observed in the Melanesian sample was the rs2228479 polymorphism. To better assess the frequency of the derived allele at this site across Northern Island Melanesia we genotyped this allele in 444 additional individuals from New Britain, New Hanover, New Ireland, and Bougainville. The frequency of the derived allele at this locus in each Melanesian island and population are reported in Table 4. The frequency of the derived allele at rs2228479 was significantly different between New Hanover and the 3 other islands sampled (Chi-squared test, all *p* < 0.001). The frequency of the derived allele also varied significantly between the Austronesian speaking (0.19) and non-Austronesian speaking (0.10) population sample (*χ*^2^ = 17.06, *p* < 0.0001).

**Table 4:** Raw mean (and S.D.) skin and hair M index values and frequency of rs2228479 derived allele in islands and neighborhoods sampled here

Island/Neighborhood	*N*	Mean skin M (SD)	Mean hair M	rs2228479 A allele frequency
Bougainville	78	89.8 (9.7)	147.6 (14.8)	0.10
**Kukuavo**	**32**	**91.9 (8.5)**	**138.6 (8.9)**	**0.12**
Saposa Island	16	82.6 (8.3)	150.1 (7.7)	0.16
New Britain	286			0.15
Arimegi Island	44	65.2 (5.3)	157.5 (11.1)	0.33
**Kariai (Anem)**	**16**	**71.3 (4.3)**	**149.0 (8.8)**	**0.09**
**Pureling (Anem)**	**12**	**67.0 (6.9)**	**146.5 (12.2)**	**0.08**
Kisiluvi (Mamousi)	29	68.8 (6.0)	155.0 (17.0)	0.05
Lingite (Mamousi)	13	69.3 (7.7)	164.4 (17.4)	0.04
“other” Mamousi	20	67.5 (4.6)	159.0 (15.7)	0.07
Loso (Nakanai)	10	70.9 (6.8)	164.3 (28.4)	0.05
**Uasilau (Ata)**	**38**	**67.4 (5.3)**	**150.6 (15.1)**	**0.09**
**Lugei (Ata)**	**11**	**67.0 (5.6)**	**173.9 (21.1)**	**0.04**
Bileki (Nakanai)	18	66.7 (6.3)	143.3 (15.4)	0.19
Ubili (Nakanai)	27	67.6 (6.5)	166.1 (20.1)	0.19
New Ireland	147	73.9 (8.4)	151.5 (22.4)	0.17
Tigak	25	72.9 (8.6)	156.0 (16.5)	0.32
Nailik	19	72.8 (6.7)	151.1 (25.1)	0.21
**Kabil (Kuot)**	**32**	**76.1 (8.3)**	**151.1 (17.0)**	**0.13**
Notsi	16	74.1 (9.9)	161.1 (21.2)	0.09
Madak	23	77.0 (8.3)	145.6 (28.3)	0.07
New Hanover	84	76.7 (7.4)	154.5 (16.2)	0.26
North Lavongai	62	76.7 (6.9)	155.2 (16.3)	0.30
South Lavongai	13	77.3 (9.9)	150.3 (14.2)	0.15

Populations in boldface type speak non-Austronesian languages

We tested for an association between genotype at rs2228479 and quantitatively measured skin and hair color using this expanded sample. Mean skin and hair values (standardized and normalized as well as raw values) are found in Table 4. Because the observed rs2228479 genotypes differed significantly from expectations under Hardy Weinberg equilibrium in the full Northern Island Melanesian sample (*p* = 3.494 × 10^−5^), possibly indicating significant population substructure, and because of previous reports of substructure in the region [48], we tested for associations in the full sample using island as a covariate as well as on each island separately. In the full sample rs2228479 is not significantly associated with variation in hair pigmentation (*p* = 0.7201). Associations with skin pigmentation are suggestive (0.0635), but not significant. These should also be interpreted with caution, since this analysis does not account for potential substructure within island groupings [48]. rs2228479 is not significantly associated with lighter skin color on any of the four islands tested, nor is it significantly associated with hair color (*p* > 0.05 for all tests).

## Discussion

The populations sampled here are all from Northern Island Melanesia, a region where UVR is high and variation in skin phenotype and at pigmentation loci is expected to be constrained by purifying selection. While pigmentation is generally dark, notable variation in both skin and hair pigmentation is observed [58], indicating the likely presence of coding or regulatory mutations in pigmentation genes. Recently an allele associated with lighter hair color in populations from the Solomon Islands was identified [59], the distribution of which is restricted to the Solomons and parts of the Bismarck Archipelago [59, 78]. This suggests that at least some of the observed variation in skin and hair phenotype in the region may be caused by population-specific alleles not currently reported in large public databases. In this paper, we set out to characterize variation in a well-known pigmentation candidate gene, *MC1R*, in a large sample of individuals from Northern Island Melanesia in order to test the following hypotheses: a) that *MC1R* variation is constrained by purifying selection in Melanesia, as it is in other high UVR populations; b) that, given this selective constraint, Melanesians should appear more closely related to other high-UVR populations (e.g., Africans) than to populations with which they share a more recent common history (other non-Africans, and specifically East Asians), and c) that nonsynonymous variants in *MC1R* are significantly associated with skin and/or hair pigmentation phenotype in Melanesian populations.

### Purifying selection

Given the high levels of UVR in Melanesia, we expected that variation in *MC1R* would be constrained in Melanesian populations, particularly in non-Austronesian speakers, as the ancestors of modern non-Austronesian speakers have been resident in the region for ~ 30 kya. Surprisingly, out of the five segregating sites that we observed in this Melanesian sample, three are nonsynonymous polymorphisms. One of these is rare (occurring at a frequency of < 1 % in the sample), while the two others, rs2228479 and rs885479, occur at frequencies of 15.4 and 4.5 % in the sequenced sample. A modified McDonald’s Kreitman’s test (suitable for tests involving a small number of segregating sites) does not support a model of purifying selection. Tajima’s D and Fay and Wu’s H values, while slightly negative, also do not indicate a significant departure from neutrality, suggesting that *MC1R* in Melanesians has not been subject to either purifying or positive selection. This absence of strong purifying selection is surprising given the high UVR levels in the region [58]. However, while we find little evidence to support strong purifying selection acting on *MC1R* in this Melanesian population sample it is likely that purifying selection has influenced variation at other pigmentation loci.

Interestingly, when we apply this modified McDonald-Kreitman’s test to the African 1000 Genomes data we also fail to reject a model of neutrality. Ten segregating sites are observed among the LWK and YRI samples, 4 of which are nonsynonymous and 6 of which are synonymous. The likelihood of observing such a pattern is *p* = 0.553. This differs from observations of Harding et al. [42], who observed only five *MC1R* haplotypes in Africans, and zero nonsynonymous polymorphisms. Using those data, the authors estimated that the probability of observing zero nonsynonymous polymorphisms out of five total polymorphisms was 0.0198, causing them to reject a model of neutral evolution in Africans. Similarly low levels of variation were observed in Africans by Rana et al. [43]. However, a subsequent study by John et al. [44] reported the presence of three nonsynonymous polymorphisms in Africans, including one (rs3212366) that occurred at a frequency of 0.11. Despite the presence of these nonsynonymous polymorphisms, however, a McDonald-Kreitman’s test indicated that variation in these African samples was constrained by purifying selection. However, an HKA test failed to reject the neutral model, and while site-frequency-spectrum based tests (e.g. Tajima’s D, Fay and Wu’s H) indicated a significant departure from neutrality, the authors noted that this could be confounded by demographic history [44]. These results demonstrate that nonsynonymous polymorphisms are present (and tolerated) in African populations (and presumably other high UVR populations. The 1000 Genome data set used here is roughly seven times the size of the dataset used in these earlier studies, which may explain the higher proportion of polymorphisms (both nonsynonymous and synonymous) that we observe. Notably, though, none of these reach a frequency greater than 0.025 in the African sample (frequency range is 0.005–0.022). This is in contrast to the nonsynonymous polymorphisms observed in the Melanesian sample, which range in frequency from 0.003 to 0.157.

### Inter-population variation

Melanesia is a region known for its extensive diversity, often characterized by high levels of inter-island phenotypic and genotypic variation [48, 57, 78, 79]. This variation can be attributed, at least in part, to the complex population history of the region [47–51]. Given these background levels of high inter-island diversity, as well as known differences in skin and hair pigmentation across islands in the region, one might expect to observe high levels of inter-island divergence at *MC1R*. However, levels of pairwise F_ST_ between islands are relatively low (0.000–0.031) suggesting that *MC1R* is not significantly differentiated among islands. This is also visualized in the Melanesian *MC1R* haplotype networks (Fig. 2), which indicate that the majority of common Melanesian *MC1R* haplotypes are shared across islands.

Constraint on pigmentation phenotype is expected to lead to lower levels of inter-population divergence at pigmentation loci between high UVR populations. In our dataset this could be visualized as reduced population differentiation (measured as F_ST_) and high levels of haplotype sharing between Melanesian and African populations. Here we observe an F_ST_ value of 0.088 between our sequenced Melanesian sample and the African populations (LWK and YRI) of the 1000 Genomes Project. Without comparisons to F_ST_ values for other genes sequenced in the same population samples it is difficult to assess whether this is unusually high or low compared to average levels of divergence between these specific populations. However, previous studies have reported the mean F_ST_ between a single Melanesian (Nasioi) and African (Mende) population to be 0.182, based on allele frequencies at ~11,000 SNPs distributed throughout the genome [11]. Other published estimates based on sequence data from non-coding autosomal regions indicate that Melanesian-African F_ST_ values range from 0.199–0.283 [80]. These suggest that while still high, the reported F_ST_ values here between Melanesians and Africans may be lower than average levels of genome-wide divergence between populations from these two regions. However, if *MC1R* function were highly conserved across high-UVR populations we would expect to observe much lower values, as well as a high degree of exclusive Melanesian-African haplotype sharing, which is not evident (see Fig. 3).

### The phenotypic role and evolutionary history of rs2228479

The derived rs2228479 allele is associated with red hair and fair skin color in European populations [36], and has been associated with lighter skin color in South Asians [13] and freckling and solar letinges in a Japanese population [61, 62]. rs2228479 also has a significant gene-gene interaction effect with the *OCA2* locus that may influence pigmentation in Tibetan populations [1]. When we typed this SNP (the only nonsynonymous mutation to reach a frequency > 5 % in our sequenced sample) in an expanded sample from across Northern Island Melanesia (total rs2228479 sample size = 635 individuals) we observe the derived allele occurring at a frequency of 16.1 % in the region. In comparison, this allele occurs at a frequency of 11-33 % in the East Asian 1000 Genomes populations, although it is generally rare in the European, African, and South Asian samples (0–12 %).

Previous studies have demonstrated that this allele results in impaired *MC1R* function relative to the human consensus sequence [81], consistent with reported associations with pigmentation phenotype. However, we find no association between rs2228479 and skin or hair color in the full sample or on any of the individual four islands that make up the bulk of the Melanesian sample used here. We have tested for associations on each island separately to avoid confounding effects of population substructure across the region (which can lead to false-positive associations). However, this results in smaller sample sizes and a loss of power to detect associations. A more robust method to control for population substructure in the larger region would be to would be to utilize genotype data from a large number of markers across the genome and conduct a PCA analyses to identify potential substructure [82, 83]. Significant PCs can then be included as covariates in association analyses.

A second possible explanation for this lack of association is that the effects of the rs2228479 are modified by epistatic interactions with other pigmentation loci [84]. This might explain the moderate frequencies of the derived rs2228479 allele in our Melanesian sample: despite the reduction in MC1R function caused by this allele, its effects are masked by other pigmentation loci and it is treated as effectively neutral. These results also indicate that polymorphisms at loci other than *MC1R* are necessary to explain the observed variation in Melanesian skin and hair pigmentation. One locus known to influence hair pigmentation in the region, rs387907171 SNP in the *TYRP1* gene [59], can explain a small proportion of hair pigmentation on these islands [78], but other, yet-to-be-identified loci also clearly play a role in shaping Melanesian pigmentation phenotype.

While epistatic effects between *MC1R* and other pigmentation loci may partially explain the frequency of the derived rs2228479 allele observed here, the demographic history of Melanesian populations may also be a contributing factor. In particular, we note the differences in the frequency of the derived rs2228479 allele between Austronesian and non-Austronesian speakers, and suggest that this allele may have been introduced to the region via Austronesian-mediated migration. Supporting this, some of the highest reported frequencies of this allele are found in East Asia, specifically in aboriginal populations from Taiwan, the putative Austronesian homeland [85], As further evidence, we note the distribution of the derived allele in Indonesian populations to the west, where it is absent from multiple non-Austronesian speaking populations, but is observed at a frequency of 0.27 in the Austronesian-speaking Biak islanders. Also relevant are the sharply decreased diversity levels of nucleotide diversity observed among the non-Austronesian individuals in our sample, which suggest that non-Austronesian *MC1R* diversity may reflect a history of long-term constraint, while the higher diversity among Austronesian speakers is indicative of a recent history outside of the region. However, most phenotypic and genetic studies indicate that sufficient admixture between the later arriving Austronesians and the descendants of the earliest inhabitants of the region has occurred to blur clear-cut distinctions between Austronesian and non-Austronesian speakers [47, 48, 58, 78, 86], and we caution against interpreting patterns of *MC1R* diversity in this sample strictly along linguistic lines. Consistent with the picture of long-term admixture, we note that haplotypes carrying the derived rs2228479 allele are observed on all islands, and in both Austronesian and non-Austronesian-speaking individuals.

## Conclusion

In this study we sequenced the *MC1R* gene in a large sample (*n* = 188) of individuals from four islands in Northern Island Melanesia, and find little indication of strong purifying selection acting on this gene. We also find little evidence of high levels of inter-island diversity in the region, contrary to other loci [48]. While F_ST_ values between our Melanesian sample and African populations sequenced in the 1000 Genomes Project indicate lower than average levels of divergence, we do not find strong evidence that ancestral *MC1R* haplotypes have been conserved in populations from these two high UVR regions. The only common nonsynonymous *MC1R* mutation observed in the Melanesian sample, rs2228479, is not associated with pigmentation phenotype in the region, possibly due to epistatic effects at other loci. These epistatic effects along with recent migrations of East Asian individuals via the Austronesian expansion can explain the observed patterns of *MC1R* diversity across the region. Pigmentation is a complex phenotype that exhibits extensive variation within and between populations, and is controlled by multiple interacting genetic loci. While *MC1R* has a strong influence on pigmentation in several non-African populations, it does not play a significant role in shaping variation in populations from Northern Island Melanesia. Clearly a better understanding of pigmentation candidate gene diversity in Melanesian populations will be necessary in order to better characterize the genetic architecture of the pigmentation phenotype in this region.

## Availability of supporting data

The DNA sequences supporting the material analyzed here are available in the GenBank repository, with accession numbers KT863240- KT863426.

## Additional files

Additional file 1:
**Histogram of untransformed and transformed values of skin M index.** (PDF 211 kb) Additional file 2:
**Histogram of untransformed and transformed values of hair M index.** (PDF 183 kb) Additional file 3: Tables S1 and S2. (DOCX 135 kb)
