# Supplementary figures and images for: MC1R diversity in Northern Island Melanesia has not been constrained by strong purifying selection and cannot explain pigmentation phenotype variation in the region
Source: BMC Genet. 2015 Oct 19;16:122. doi: 10.1186/s12863-015-0277-x (PMC4615358; doi:10.1186/s12863-015-0277-x)

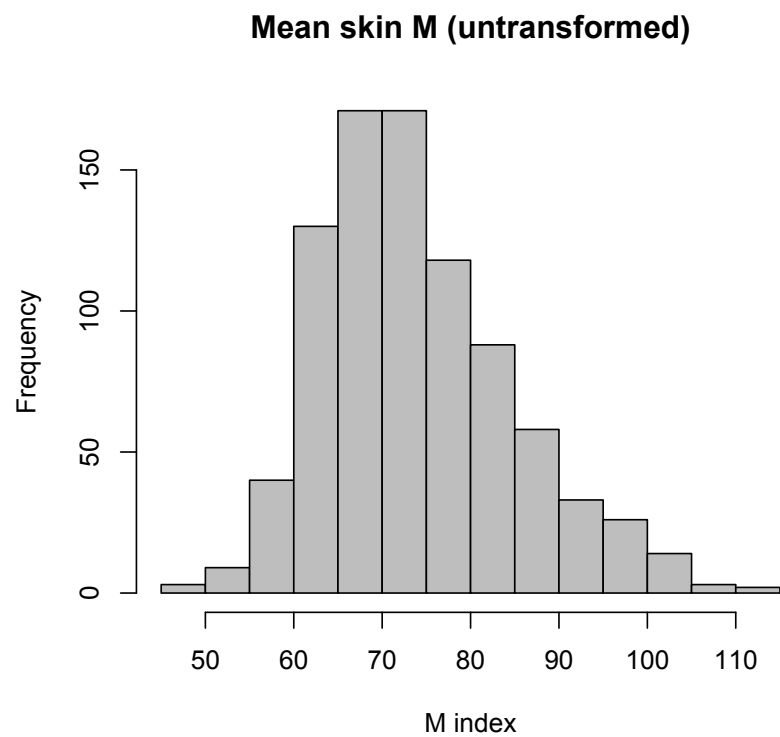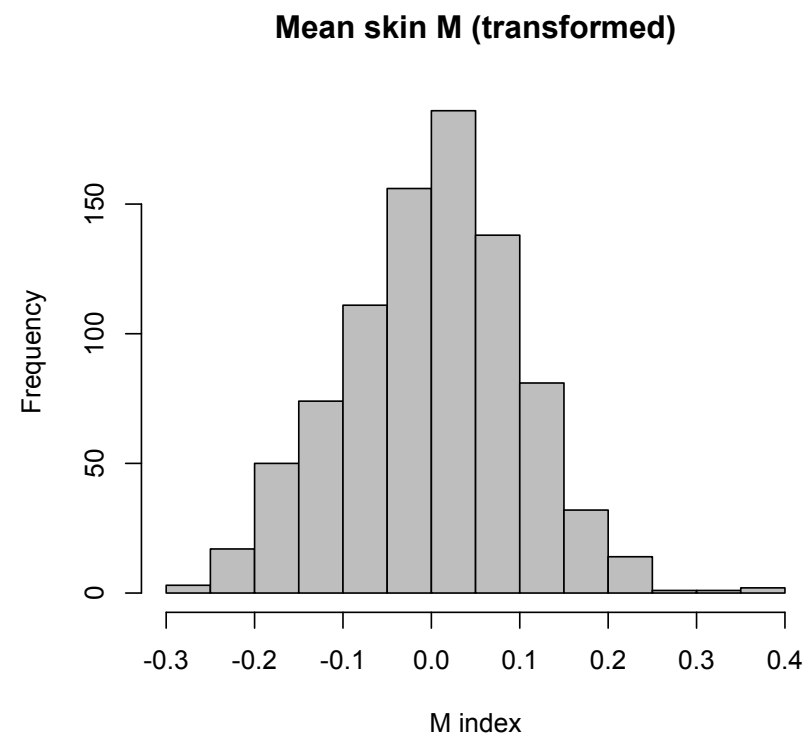

Additional Figure 1: Untransformed and transformed values of skin M index.

Supplement: Additional file 1: — Histogram of untransformed and transformed values of skin M index. (PDF 211 kb) [file 12863_2015_277_MOESM1_ESM.pdf]

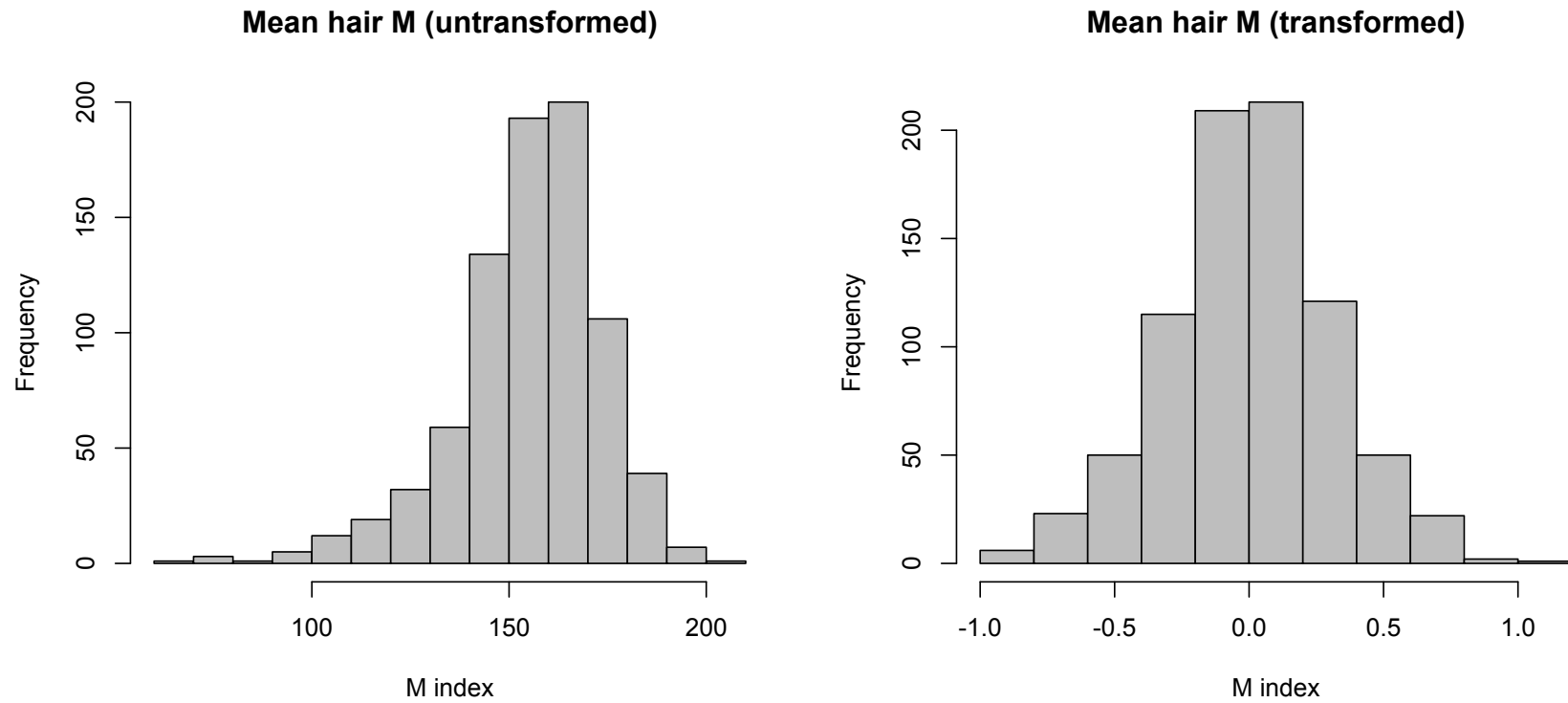

Supplementary Figure 2: Untransformed and transformed values of hair M index.

Supplement: Additional file 2: — Histogram of untransformed and transformed values of hair M index. (PDF 183 kb) [file 12863_2015_277_MOESM2_ESM.pdf]
