# Supplementary material for: MC1R diversity in Northern Island Melanesia has not been constrained by strong purifying selection and cannot explain pigmentation phenotype variation in the region
Source: BMC Genet. 2015 Oct 19;16:122. doi: 10.1186/s12863-015-0277-x (PMC4615358; doi:10.1186/s12863-015-0277-x)
Supplement: Additional file 3: Tables S1 and S2. — (DOCX 135 kb) [file 12863_2015_277_MOESM3_ESM.docx]

| Population | N | π (observed) | θ (observed) | Mean π over 100 subsamples of 370 chromosomes(range) | Mean θ over 100 subsamples of 370 chromosomes (range) | π rank percentile | θ rank percentile |
| --- | --- | --- | --- | --- | --- | --- | --- |
| AFR | 370 | 0.00086 | 0.00162 | NA | NA | NA | NA |
| EAS | 572 | 0.00138 | 0.00121 | 0.00137 (0.00126-0.00147) | 0.00111 (0.00081-0.00129) | 0.50 | 0.26 |
| EUR | 758 | 0.00104 | 0.00247 | 0.00104 (0.00092-0.00115) | 0.00229 (0.00178-0.00275) | 0.53 | 0.09 |
| MEL | 376 | 0.00075 | 0.00081 | 0.00039 (0.00039-0.00040) | 0.00065 (0.00065-0.00065) | 0.00 | 0.00 |
| SAS | 978 | 0.00061 | 0.00323 | 0.00061 (0.00053-0.00071) | 0.00178-0.00323 (0.00234) | 0.60 | 0.01 |

Additional Table 1: Observed and subsampled values of π and θ from EAS, EUR, MEL, and SAS populations subsampled down to 370 chromosomes each. Each population was subsampled 100 times, and the mean and range of π and θ for each population are reported. The rank percentile of the observed π and θ relative to the subsampled dataset is determined as rank percentile(x) = # of subsampled datasets ≥ observed statistic/100 simulations.

| NH | 0.030 |  |  |  |  |  |  |  |  |  |  |  |  |  |  |  |  |  |
| --- | --- | --- | --- | --- | --- | --- | --- | --- | --- | --- | --- | --- | --- | --- | --- | --- | --- | --- |
| NB | 0.005 | 0.042 |  |  |  |  |  |  |  |  |  |  |  |  |  |  |  |  |
| NI | 0.000 | 0.031 | 0.000 |  |  |  |  |  |  |  |  |  |  |  |  |  |  |  |
| LWK | 0.088 | 0.090 | 0.118 | 0.092 |  |  |  |  |  |  |  |  |  |  |  |  |  |  |
| YRI | 0.086 | 0.089 | 0.116 | 0.090 | 0.000 |  |  |  |  |  |  |  |  |  |  |  |  |  |
| CEU | 0.069 | 0.112 | 0.043 | 0.057 | 0.167 | 0.164 |  |  |  |  |  |  |  |  |  |  |  |  |
| FIN | 0.080 | 0.111 | 0.032 | 0.058 | 0.174 | 0.172 | 0.010 |  |  |  |  |  |  |  |  |  |  |  |
| GBR | 0.037 | 0.060 | 0.023 | 0.030 | 0.122 | 0.122 | 0.007 | 0.019 |  |  |  |  |  |  |  |  |  |  |
| IBS | 0.108 | 0.175 | 0.087 | 0.098 | 0.220 | 0.215 | 0.049 | 0.065 | 0.042 |  |  |  |  |  |  |  |  |  |
| TSI | 0.056 | 0.105 | 0.047 | 0.052 | 0.156 | 0.154 | 0.024 | 0.042 | 0.012 | 0.001 |  |  |  |  |  |  |  |  |
| CHB | 0.302 | 0.237 | 0.233 | 0.266 | 0.316 | 0.312 | 0.251 | 0.184 | 0.221 | 0.351 | 0.295 |  |  |  |  |  |  |  |
| CHD | 0.291 | 0.199 | 0.238 | 0.261 | 0.292 | 0.289 | 0.273 | 0.216 | 0.227 | 0.371 | 0.306 | 0.025 |  |  |  |  |  |  |
| JPT | 0.425 | 0.357 | 0.341 | 0.382 | 0.428 | 0.421 | 0.333 | 0.251 | 0.307 | 0.449 | 0.388 | 0.022 | 0.089 |  |  |  |  |  |
| BEB | 0.018 | 0.072 | 0.011 | 0.010 | 0.077 | 0.077 | 0.058 | 0.053 | 0.041 | 0.093 | 0.057 | 0.258 | 0.265 | 0.361 |  |  |  |  |
| GIH | 0.005 | 0.064 | 0.020 | 0.007 | 0.064 | 0.064 | 0.067 | 0.078 | 0.046 | 0.101 | 0.058 | 0.303 | 0.301 | 0.417 | 0.004 |  |  |  |
| ITU | 0.018 | 0.081 | 0.018 | 0.014 | 0.080 | 0.079 | 0.059 | 0.062 | 0.044 | 0.084 | 0.053 | 0.286 | 0.294 | 0.392 | 0.000 | 0.001 |  |  |
| PJL | 0.004 | 0.061 | 0.009 | 0.003 | 0.073 | 0.073 | 0.060 | 0.064 | 0.038 | 0.088 | 0.050 | 0.287 | 0.288 | 0.400 | 0.001 | 0.000 | 0.000 |  |
| STU | 0.017 | 0.085 | 0.021 | 0.015 | 0.079 | 0.078 | 0.062 | 0.069 | 0.048 | 0.095 | 0.060 | 0.301 | 0.307 | 0.411 | 0.001 | 0.000 | 0.000 | 0.000 |
|  | B | NH | NB | NI | LWK | YRI | CEU | FIN | GBR | IBS | TSI | CHB | CHD | JPT | BEB | GIH | ITU | PJL |

Additional Table 2: Pairwise F_ST_ between individual Melanesian and 1KG populations. B: Bougainville; NH: New Hanover; NB: New Britain; NI: New Ireland; LWK: Luhya; YRI: Yoruba; CEU: CEPH from Utah; FIN: Finn; GBR: Great Britain; IBS: Iberians from Spain; TSI: Tuscans from Italy; CHB: Han Chinese from Beijing; CHD: Han Chinese from Denver; JPT: Japanese from Tokyo; BEB: Bengali from Bangladesh; GIH: Gujarati Indians from Houston, TX; ITU: Indian Telugu from the UK; PJL: Punjabi from Lahore, Pakistan; STU: Sri Lankan Tamil from the UK.
